# Gelatin and Antioxidant Peptides from Gelatin Hydrolysate of Skipjack Tuna (*Katsuwonus pelamis*) Scales: Preparation, Identification and Activity Evaluation

**DOI:** 10.3390/md17100565

**Published:** 2019-10-03

**Authors:** Yi-Ting Qiu, Yu-Mei Wang, Xiu-Rong Yang, Yu-Qin Zhao, Chang-Feng Chi, Bin Wang

**Affiliations:** 1 Zhejiang Provincial Engineering Technology Research Center of Marine Biomedical Products, School of Food and Pharmacy, Zhejiang Ocean University, Zhoushan 316022, China; qytdezh@icloud.com (Y.-T.Q.); wangym731@126.com (Y.-M.W.); zhaoy@hotmail.com (Y.-Q.Z.); 2 National and Provincial Joint Laboratory of Exploration and Utilization of Marine Aquatic Genetic Resources, National Engineering Research Center of Marine Facilities Aquaculture, School of Marine Science and Technology, Zhejiang Ocean University, Zhoushan 316022, China; yxr1948008999@163.com

**Keywords:** skipjack tuna (*Katsuwonus pelamis*), scale, gelatin, peptide, antioxidant activity

## Abstract

For full use of fish by-products, scale gelatin (TG) and antioxidant peptides (APs) of skipjack tuna (*Katsuwonus pelamis*) were prepared, and their properties were characterized using an amino acid analyzer, sodium dodecyl sulfate-polyacrylamide gel electrophoresis (SDS-PAGE), Fourier transform infrared spectroscopy (FTIR), electrospray ionization mass spectrometers (ESI-MS), and radical scavenging assays. The results indicate that TG with a yield of 3.46 ± 0.27% contained Gly (327.9 ± 5.2 residues/1000 residues) as the major amino acid and its imino acid content was 196.1 residues/1000 residues. The structure of TG was more unstable than that of type I collagen from scales of skipjack tuna (TC) and TG was more suitable for preparation of hydrolysate by protease than mammalian gelatins. Therefore, TG was separately hydrolyzed under five proteases (pepsin, papain, trypsin, neutrase, and alcalase) and ten APs (TGP1–TGP10) were isolated from the alcalase-hydrolysate. Among them, TGP5, TGP7, and TGP9 with high antioxidant activity were identified as His-Gly-Pro-Hyp-Gly-Glu (TGP5), Asp-Gly-Pro-Lys-Gly-His (TGP7) and Met-Leu-Gly-Pro-Phe-Gly-Pro-Ser (TGP9), respectively. Furthermore, TGP5, TGP7, and TGP9 exhibited a high radical scavenging capability on 2,2-diphenyl-1-picrylhydrazyl (DPPH) radical (EC_50_ values of 1.34, 0.54, and 0.67 mg/mL, respectively), hydroxyl radical (EC_50_ values of 1.03, 0.41, and 0.74 mg/mL, respectively), and superoxide anion radical (EC_50_ values of 1.19, 0.71, and 1.59 mg/mL, respectively). These results suggest that three APs (TGP5, TGP7, and TGP9), especially TGP7, have a strong antioxidant activity and could act as potential antioxidant ingredients applied in functional products.

## 1. Introduction

Gelatin is a denatured form of collagen and traditionally extracted by processing by-products of land mammals, such as beef bones, bovine hides, and pig skins [1,2,3]. According to the acid and alkali preparation processes, the produced gelatins were divided into type A and type B, with a molecular weight (MW) ranging from 80 to 250 kDa [3,4]. Moreover, acid or alkali with heating treatments led to the structure of gelatin being transited from helix to coli form and significantly increased the water solubility of gelatin [4,5]. Presently, more than 450 kilotons of gelatins are required to apply in food, nutraceuticals, beverage, pharmaceuticals, cosmetic, and photographic industries, and approximately 98.5% of the global commercial gelatins are extracted from mammalian species, such as pigs and cows [1,3,6]. Nevertheless, religious sentiments and the anxiety of consumers on the safety of mammalian gelatins have led to producers needing to find new alternative sources of mammalian gelatins [1,7]. Therefore, gelatins derived from fish by-products are considered to be a promising alternative to mammalian gelatins due to their production, safety, and non-religious conflict [4,8]. In addition, fish by-products can pollute the environment if they are discarded as wastes. Therefore, the application of fish by-products for fish gelatin preparation can effectively reduce environmental pollution and the produced gelatins can be used in food products and increase the income of fish industries [3].

At present, fish gelatin has been extensively extracted from different fish by-products, such as shark (*Isurus oxyrinchus*) cartilages [9], swim bladders of yellowfin tuna [10], scales of bighead carp (*Hypophthalmichthys nobilis*) [11] and Lizardfish [12], skins of unicorn leatherjacket [13] and European eel (*Anguilla anguilla*) [14], and bones of skipjack tuna (*Katsuwonus pelamis*), red snapper and grouper [4,15]. Compared with mammalian gelatins, fish gelatins have a lower content of imino acids (Pro and Hyp), which weakens their structural stability and rheological behaviors, lowers their melting temperature and gel strength [3,4]. Those physicochemical characteristics greatly limit their applications as tissue engineering materials, but fish gelatins are more suitable for preparation of bioactive peptides because they are more easily hydrolyzed by protease due to their weak structural specialty [4]. Even more exciting is the fact that many bioactive peptides derived from fish gelatins show strong biological activities and have attracted wide attention due to their broad application potentiality in biomedical and health care industries. Mendis *et al.* reported that HGPLGPL from gelatin hydrolysate of Hoki skin could serve as an antioxidant against linoleic acid peroxidation, and its activity was closer to the highly active synthetic antioxidant of butylated hydroxytoluene [16]. Ngo et al. reported that MVGSAPGVL and LGPLGHQ from skin gelatin hydrolysate of skate (*Okamejei kenojei*) could protect human endothelial cells from oxidative damage by increasing the expression levels of antioxidant factors (superoxide dismutase (SOD) and glutathione (GSH)) in EA.hy926 cells [17]. GGFDMG from the skin gelatin hydrolysate of Japanese flounder can protect the radical-mediated damage of membrane lipids, proteins, and DNA by upregulating the expression of inherent antioxidative enzymes (SOD, GSH, and catalase (CAT)) [18]. Similar results were reported that skin gelatin hydrolysate of Pacific cod could protect the skin from UV radiation-induced damage by regulating the level of endogenous antioxidant enzymes and inhibiting nuclear factor-κB (NF-κB) expression [19], and two interstitial collagenase (MMP-1) inhibitory peptides (GEIGPSGGRGKPGKDGDAGPK and GFSGLDGAKGD) isolated from the hydrolysate, showed a significant inhibition on MMP-1, phosphorylation of extracellular-signal-regulated kinase (p-ERK) and phospho-p38 (p-p38). Furthermore, GEIGPSGGRGKPGKDGDAGPK could significantly inhibit phospho-Jun N-terminal kinase (p-JNK) in mitogen-activated protein kinase (MAPK) signaling pathways [20]. Those studies suggested that fish gelatin peptides have great potential to serve as antioxidants in healthcare foods due to their capability to regulate intracellular antioxidant defense systems and signaling pathways of MAPK and NF-κB.

As an important commercial species, skipjack tuna (*K. pelamis*) supports a growing global production of more than 3 million t/year in 2016 [21]. In China, large amounts of fish byproducts, including scales, skins, heads, and viscera are produced during the processing of tuna cans production [22], and partial bioactive compounds including collagens, gelatins, and bioactive peptides were prepared from those bones, heads, and black muscles [4,22,23]. This is regarded as the most reasonable approach to process by-products of skipjack tuna. However, there is no information available about the preparation of gelatin and bioactive peptides using skipjack tuna scales. Therefore, the aims of this study were to prepare and characterize gelatin (TG) from skipjack tuna scales, isolate and identify the APs from the gelatin hydrolysate, and evaluate the radical scavenging activities of isolated APs. 

## 2. Results and Discussion

### 2.1. Characterization of Scale Gelatin (TG) of Skipjack Tuna

#### 2.1.1. Proximate Composition and Yield of TG

As shown in Table 1, the yield of TG was 3.46 ± 0.27% (on a dry scale weight basis), which was significantly lower than those of gelatins from skins (11.3 ± 0.03%) [24] and bones (6.37 ± 0.64%) [4] of skipjack tuna. The protein content of TG was 94.08 ± 4.52 g/100 g, which was significantly higher than the contents of moisture (3.78 ± 0.39%), fat (0.53 ± 0.22%), and ash (1.05 ± 0.16) in the TG. In addition, the protein content of TG was significantly higher than those of gelatins from the skins (88.4 ± 0.12 g/100 g) and bones (90.14 ± 3.98 g/100 g) of skipjack tuna [25].

#### 2.1.2. Amino Acid Composition of TG

As shown in Table 2, the amino acid pattern of gelatin (TG) from the scales of skipjack tuna was similar to that of type I collagen from scales of skipjack tuna (TC). Glycine (Gly) was the highest content of amino acid of TG and TC with contents of 327.9 ± 5.2 and 330.6 ± 4.6 residues/1000 residues, respectively. The reason is that about 50–60% of α-chains in collagen are composed of typical tripeptide repetitions (Gly-X-Y) [26,27]. In addition, TG and TC were rich in alanine (Ala), proline (Pro), and hydroxyproline (Hyp) with a descending order. The amino acid pattern of gelatin (TG) was similar to those of gelatins from bovine heart [28] and skin [5], tuna bones and skin [4,24], and salmon, rohu and shark skins [24,29].

The amount of imino acids (Pro and Hyp) is closely related to the stability of the gelatins and collagens because the pyrrolidine rings of Pro and Hyp can assist in maintaining the stability of the triple helical structure [4,30]. In addition, the hydroxyl group in Hyp can form intermolecular hydrogen bonds to reinforce the triple-stranded helix of gelatins [4,24]. Table 2 shows that the content of imino acids of TG was 196.1 residues/1000 residues, which is slightly higher than those of gelatins from dover sole skins (173–183 residues/1000 residues) [31], tuna bones (177.3 residues/1000 residues) [4], and bigeye snapper skins (186–187 residues/1000 residues) [32], but significantly lower than that of bovine gelatin (219.0 residues/1000 residues) [33]. The data indicate that the helices of TG were more unstable and easier to hydrolysis than bovine gelatin.

#### 2.1.3. Electrophoretic Pattern of TG

**Figure 1:**
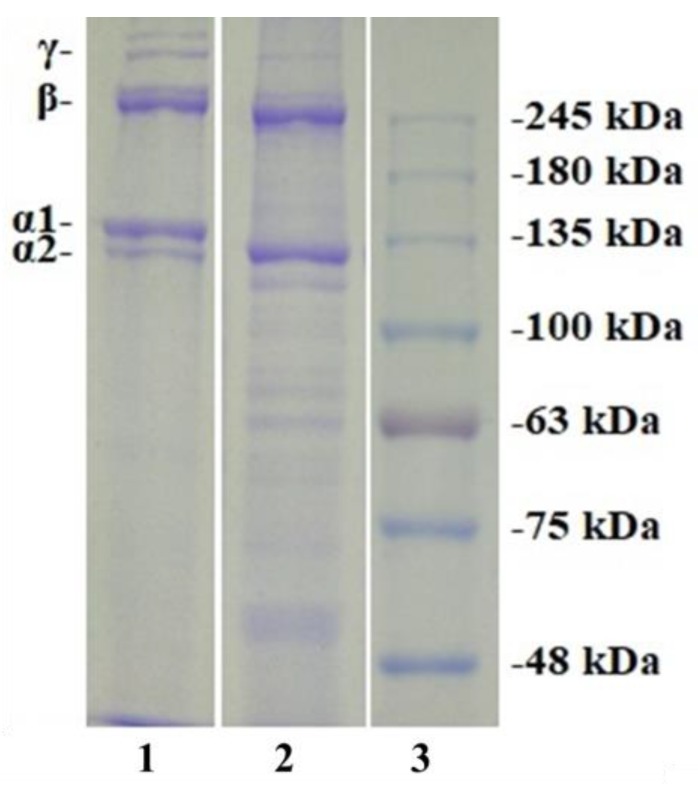
SDS-PAGE patterns of scale gelatin (TG) and type I collagen (TC) of skipjack tuna analyzed by 7.5% separating gel and 4% stacking gel. Lane 1. TC; Lane 2. TG; Lane 3. Protein marker.

The MW distribution and subunit compositions can significantly affect the properties of gelatins and collagens. Figure 1 presents the sodium dodecyl sulfate-polyacrylamide gel electrophoresis (SDS-PAGE) patterns of TG and TC. The image indicates that TG had similar protein patterns to those of TC. They were composed of two α-chains (α1 and α2 chains with the band intensity ratio of 2:1). The pattern of TG ([α1]_2_α2) was similar to those of gelatins from seafood by-products, such as the bones of skipjack tuna [4] and the skins of shark, rohu, tuna [24] and carp (*Cyprinus carpio*) [30]. In addition, β-chain (α-chain dimer) were also found in the protein patterns of TG and TC, but the band intensities of γ chain (α-chain trimer) of TC were significantly stronger than those of TG. A series of peptide fragments with MW below 100 kDa was noticeable in the protein patterns of TG. These results indicate that partial triple helical structure and peptide bonds of TG were degraded during the heating extraction process [4,34].

#### 2.1.4. Fourier Transform Infrared Spectroscopy (FTIR) of TG

**Figure 2:**
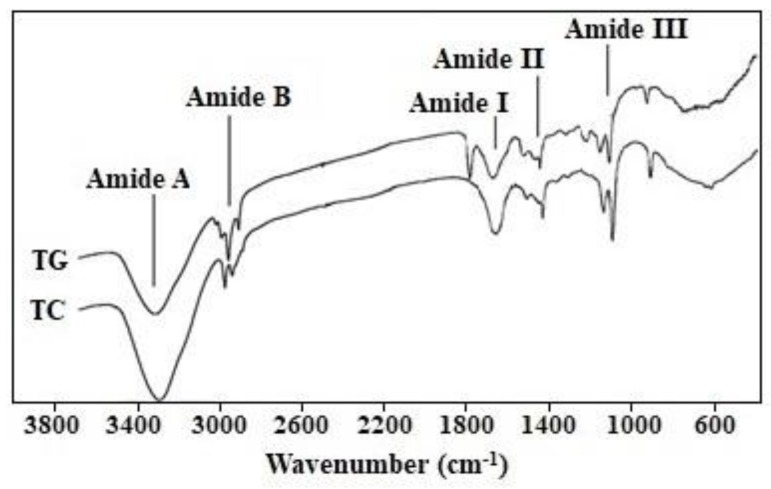
Fourier transform infrared spectra of scale gelatin (TG) and type I collagen (TC) of skipjack tuna.

The FTIR spectral profiles of TG and TC are shown in Figure 2 and the wavenumber of five major peaks (Amide A, B and I-III) are presented in Table 3. The spectrum of TG was similar to those of TC and gelatins from bones of skipjack tuna [4] and golden carp skins [35].

Amide I, II, and III bands are caused by C=O stretching, N–H bending, and C–H stretching, respectively. They are bound up with the triple helical structures of gelatins [4,35]. A lower wavenumber amide I bands (1600–1700 cm^−1^) indicates a reduction of the gelatin molecular order [35,36]. The wavenumber of TG (1667 cm^−1^) was lower than that of TC (1689 cm^−1^), which suggests that a fraction of telopeptides of TG were hydrolyzed during the heating preparation process. A low wavenumber of the amide II band suggests that gelatin has a higher structure order because it forms more hydrogen bonding by N–H groups [26,36]. The wavenumber of TG was found to be 1536 cm^−1^, which was higher than that of TC (1484 cm^−1^), which suggests that TG has less hydrogen bonding than TC. The wavenumber of amide III band is related to the triple helix structure of gelatin. In Figure 2, the amide III bands of TG and TC are located at wavenumbers of 1215 cm^−1^ and 1209 cm^−1^, respectively. The data suggests that more α-helix structure was converting to random coils upon heating extracting process. The wavenumber of free N–H groups (3400–3440 cm^−1^) was removed to a lower frequency, indicating the N–H group of the peptide involved in hydrogen bonding [4,35]. Figure 2 shows that the amide A wavenumbers of TC (3325 cm^−1^) and TG (3351 cm^−1^) suggest that the degree of hydrogen bonding in TC was more than that of TG. The amide B band is related to the asymmetric stretch vibrations of –NH_3_^+^ and =C–H, and the high wavenumber of amide B band indicates an increase in free –NH_3_^+^ groups. The wavenumbers of the amide B of TG (2939 cm^−1^) were lower than that of TC (2977 cm^−1^). The data suggests that TG has freer –NH_3_^+^ groups than TC because it formed fewer hydrogen bonds compared with TC. 

Based on the results of amino acid composition, SDS-PAGE patterns, and FTIR spectra of TG, we found that the structure of TG was more unstable than TC. Therefore, TG should be suitable to prepare active peptides because of its unstable structure.

### 2.2. Purification of APs from TG Hydrolysate

#### 2.2.1. Preparation of TG Hydrolysate

As shown in Table 4, TG was separately hydrolyzed under five kinds of proteases, and the alcalase-hydrolysate (TGH) showed the highest degree of hydrolysis (DH) (25.35 ± 1.68%) and HO· scavenging activity (29.46 ± 1.37%) at a concentration of 5.0 mg/mL among five kinds of hydrolysates. The specificity of protease serving for the hydrolysis process is the most important factor of the preparation of biopeptides [14]. As shown in Table 4, the protein hydrolysates revealed significantly different DH and antioxidant activities, mainly due to the various spectra of hydrolysate specificities, such as chain length, amino acid sequence, and spatial structure [14,37]. As an endo-protease, alcalase showed a strong proteolytic activity and was wildly used in hydrolyzing seafoods and their byproducts, such as Antartic krill [38], tuna backbone, black muscle and heads [23,39,40], sardinelle heads and viscera [41], and croceine croaker muscle [42]. In this experiment, TGH exhibited the highest radical scavenging activity and was chosen for the following separation process of APs.

#### 2.2.2. Purification of APs from TGH

**Figure 3:**
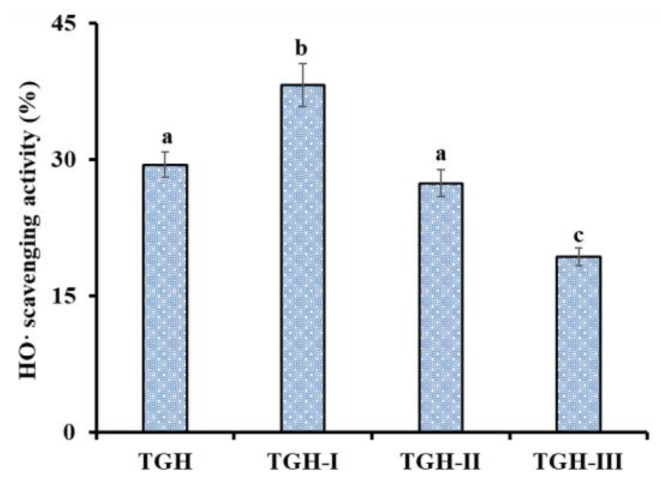
HO· scavenging activity (%) of TGH and its three fractions (TGH-I, TGH-II, and TGH-III). The data are expressed as mean ± SD (*n* = 3). ^a–c^ The values with the same letters indicate no significant difference (*p* > 0.05).

Using 3 and 5 kDa ultrafiltration membranes, three fractions, including TGH-I (<3 kDa), TGH-II (3–5 kDa), and TGH-III (>5 kDa), were prepared from TGH and their HO· scavenging activity are shown in Figure 3. The HO· scavenging activity of TGH-I was 38.19 ± 2.35% at a concentration of 5.0 mg/mL, which was significantly higher than those of TGH (29.46 ± 1.37 mg/mL), TGH-II (27.42 ± 1.46%), and TGH-III (19.36 ± 0.97%) (*p* <0.05). The lowest MW fraction (TGH-I) having the strongest activity is consistent with previous reports that the antioxidant capability of hydrolysates was inversely related to their average MW because they were more easily contacted with free radicals [2,43]. Then, TGH-I was further purified using chromatographic methods.

**Figure 4:**
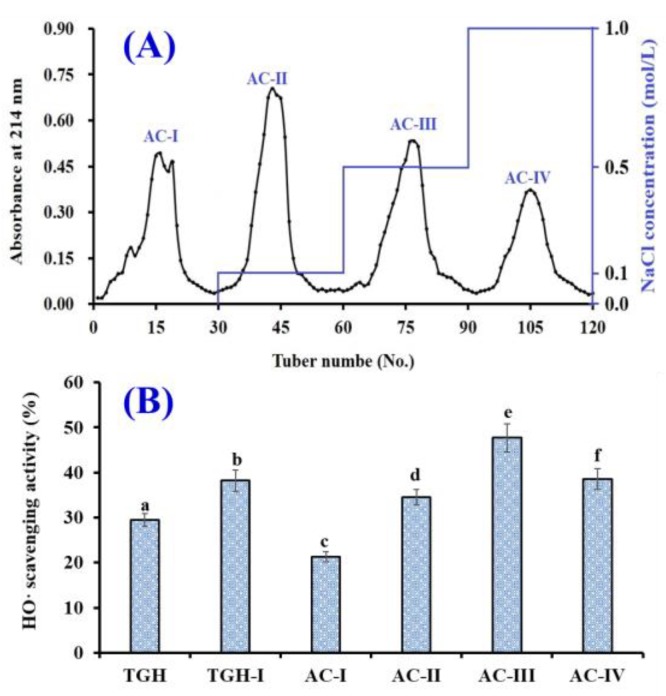
Elution profile of TGH-I in DEAE-52 cellulose anion-exchange chromatography (**A**) and HO· scavenging activity (%) of TGH-I and its four fractions (AC-I to AC-IV) (**B**). The data are expressed as mean ± SD (*n* = 3). ^a–f^ The values with same letters indicate no significant difference (*p* > 0.05).

As shown in Figure 4A, four fractions (AC-I to AC-IV) were separated from TGH-I using a DEAE-52 cellulose column. The HO· scavenging activity of AC-III was 47.72 ± 3.11% at a concentration of 5.0 mg/mL, which was significantly stronger than those of TGH (29.46 ± 1.37%), TGH-I (38.19 ± 2.35%), AC-I (21.34 ± 1.14%), AC-II (34.53 ± 1.68%), and AC-IV (38.56 ± 2.31%) (*p* <0.05) (Figure 4B). Thus, AC-III was selected for the following experiment.

**Figure 5:**
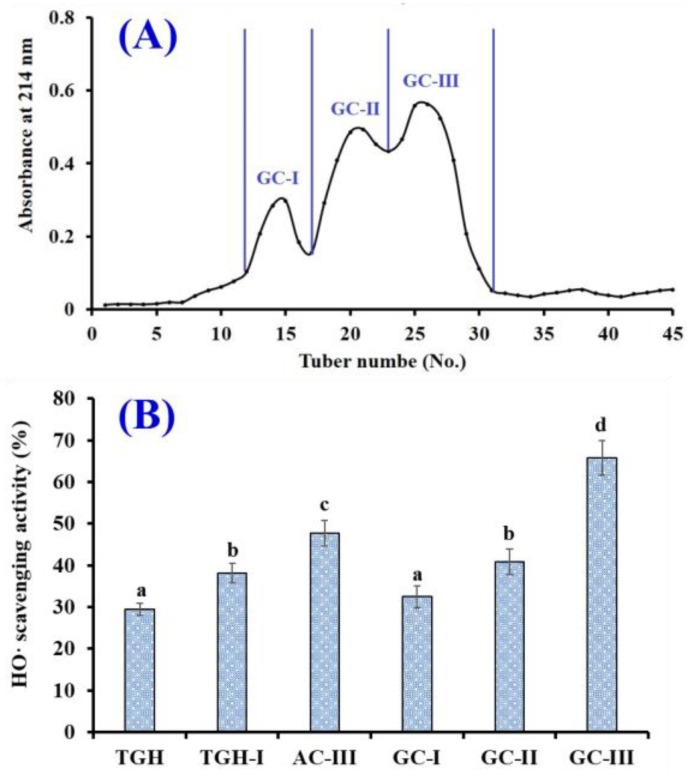
The elution profile of AC-III in Sephadex G-25 chromatography (**A**) and the HO· scavenging activity (%) of AC-III and its three fractions (GC-I to GC-III) (**B**). The data are expressed as mean ± SD (*n* = 3). ^a–d^ The values with same letters indicate no significant difference (*p* > 0.05).

As shown in Figure 5A, AC-III was further divided into three fractions (GC-I to GC-III) using a Sephadex G-25 column. The HO· radical scavenging activity of GC-III was 65.79 ± 4.21% at a concentration of 5.0 mg/mL, which was significantly stronger than those of TGH (29.46 ± 1.37%), TGH-I (38.19 ± 2.35%), AC-III (47.72 ± 3.11%), GC-I (32.46 ± 2.65%), and GC-II (40.78 ± 3.04%) (*p* < 0.05) (Figure 4B). Then, GC-III was selected for the following reversed-phase high-performance liquid chromatography (RP-HPLC) separation process. 

**Figure 6:**
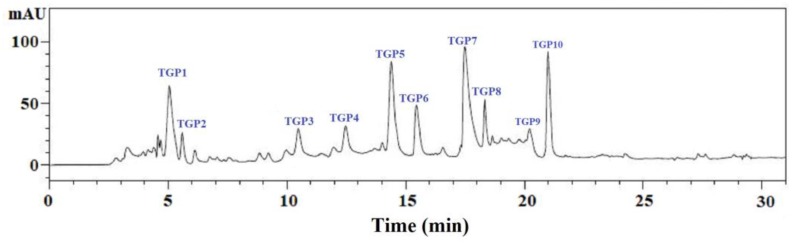
Elution profile of GC-III separated by the reversed-phase high-performance liquid chromatography (RP-HPLC) on a Zorbax, SB C-18 column (4.6 mm × 250 mm) from 0 to 30 min.

As shown in Figure 6, GC-III was finally purified using the RP-HPLC system with a Zorbax C-18 column, and the eluted peptides were gathered separately on their chromatographic peaks. At last, ten peptides with retention times of 5.006 min (TGP1), 5.067 min (TGP2), 10.535 min (TGP3), 12.612 min (TGP4), 14.421 min (TGP5), 15.436 min (TGP6), 17.593 min (TGP7), 18.186 min (TGP8), 20.215 min (TGP9), and 21.009 min (TGP10) were prepared for an HO· radical scavenging activity evaluation.

**Figure 7:**
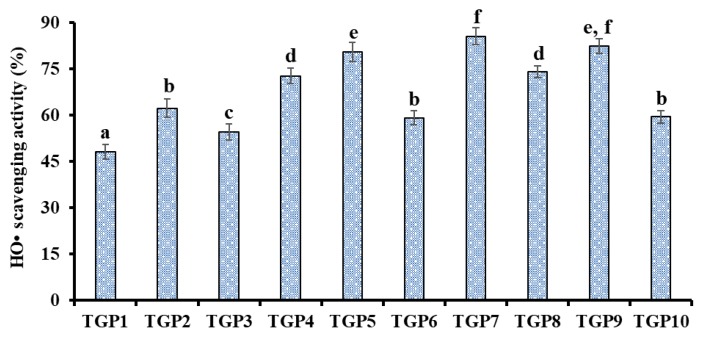
HO· scavenging activity of ten major sub-fractions (TGP1 to TGP10) of GC-III at the concentration of 5.0 mg/mL. The data are presented as the mean ± SD (*n* = 3). ^a–f^ The column wise values with same superscripts indicate no significant difference (*p* > 0.05).

Figure 7 shows the HO· scavenging activity of ten isolated APs (TGP1 to TGP10). At a concentration of 5.0 mg/mL, the HO· scavenging activities of TGP5, TGP7, and TGP9 were 80.51 ± 3.05%, 85.66 ± 2.68%, and 82.41 ± 2.34%, respectively, which is significantly higher than those of the other seven peptides. Therefore, TGP5, TGP7, and TGP9 were chosen for an amino acid sequence analysis.

### 2.3. Amino Acid Sequence and MW Analysis

The amino acid sequences of TGP5, TGP7, and TGP9 were determined using a protein sequencer and identified as His-Gly-Pro-Hyp-Gly-Glu (TGP5), Asp-Gly-Pro-Lys-Gly-His (TGP7), Met-Leu-Gly-Pro-Phe-Gly-Pro-Ser (TGP9), respectively. Using electrospray ionization mass spectrometers (ESI-MS), the average MWs of TGP5, TGP7, and TGP9 were determined as 608.57, 609.61, and 804.92 Da, respectively, which agreed well with their theoretical masses (Table 5).

### 2.4. Antioxidant Activity

#### 2.4.1. Radical Scavenging Activity

##### DPPH· Scavenging Activity

**Figure 8:**
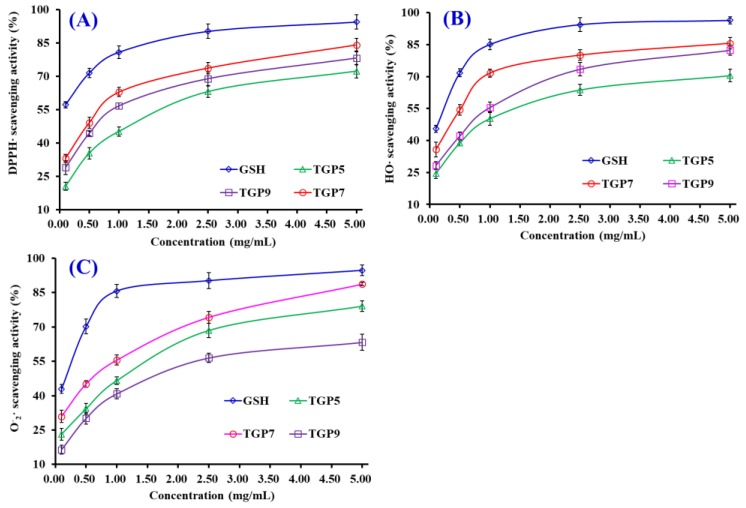
DPPH· (**A**), HO· (**B**), and $O_{2}^{-}$· (**C**) scavenging activity of three APs (TGP5, TGP7, and TGP9) from gelatin hydrolysate of skipjack tuna (*K. pelamis*) scales. Glutathione (GSH) was used as the positive control. The data are presented as the mean ± SD (*n* = 3).

Figure 8A shows that three APs (TGP5, TGP7, and TGP9) could positively scavenge DPPH· at a concentration range of 0.1–5.0 mg/mL, but their activity was lower than that of the positive control of glutathione (GSH). The EC_50_ value of TGP7 was 0.54 mg/mL, which is significantly lower than those of TGP5 (1.34 mg/mL), TGP9 (0.67 mg/mL), and partial APs from protein hydrolysates of skate cartilages (FIMGPY: 2.60 mg/mL; GPAGDY: 3.48 mg/mL; IVAGPQ: 3.93 mg/mL) [44], skipjack tuna heads (VEE: 3.76 mg/mL; DAGPYGPI: 1.33 mg/mL; ERGPLGPH: 0.93 mg/mL) [4], salmon pectoral fin (TTANIEDRR: 2.50 mg/mL) [45], miiuy croaker muscles (TWKVV: 2.67 mg/mL; VIAPW: 0.96 mg/mL) [46] and swim bladders (GFYAA: 5.02 mg/mL; FSGLR: 4.01 mg/mL; GIEWA: 0.78 mg/mL) [47]. Therefore, TGP7 had a powerful capability to hold back the DPPH· reaction through donating hydrogens or clear free radicals.

##### HO· Scavenging Activity

Figure 8B indicates that three APs (TGP5, TGP7, and TGP9) showed dose-related effects in the scavenging activity of HO· when the peptide concentrations were within the scope of 0.1 to 5.0 mg/mL. The EC_50_ values of TGP5, TGP7, and TGP9 were 1.03, 0.41, and 0.74 mg/mL, respectively. TGP7 exhibited the highest HO· scavenging capability among three APs. In addition, the EC_50_ values of TGP7 were lower than those of most APs from protein hydrolysates of skipjack tuna heads (VEE: 2.43 mg/mL; DAGPYGPI: 1.71 mg/mL; ERGPLGPH: 0.81 mg/mL) [4], grass carp skins (VGGRP: 2.06 mg/mL; PYSFK: 2.28 mg/mL) [48], miiuy croaker muscles (TWKVV: >5.00 mg/mL; VIAPW: 1.31 mg/mL) [46] and swim bladders (GFYAA: 2.35 mg/mL; FSGLR: 2.45 mg/mL; GIEWA: 0.71 mg/mL) [47]. Nevertheless, the activity of TGP7 was still lower than that of GSH. HO· can unselectively oxidize biomacromolecules and initiate the process of oxidative stress in an organism. The present data suggests that TGP7 could act as a HO· scavenger to eliminate its damage in biological systems. 

##### $O_{2}^{-}$· Scavenging Activity

Figure 8C demonstrates that three APs (TGP5, TGP7, and TGP9) showed strong $O_{2}^{-}$· scavenging activities with EC_50_ values of 1.19, 0.71, and 1.59 mg/mL, respectively. The EC_50_ values of TGP7 were lower than those of APs from protein hydrolysates of skipjack tuna heads (VEE: 1.79 mg/mL; DAGPYGPI: 1.51 mg/mL; ERGPLGPH: 3.04 mg/mL) [4], skate cartilages (IVAGPQ: 1.82 mg/mL) [44], Spanish mackerel skins (PFGPD: 0.91 mg/mL; PYGAKG: 0.80 mg/mL) [49] and miiuy croaker muscles (NFWWP: 0.84 mg/mL; TWKVV: 0.73 mg/mL; YFLWP: 3.08 mg/mL; WVWWW: >5.00 mg/mL) [46] and swim bladders (FYKWP: 1.92 mg/mL; GFYAA: 3.03 mg/mL; VPDDD: 4.11 mg/mL; FSGLR: 3.35 mg/mL) [47]. $O_{2}^{-}$· can render into the highly reactive HO· to initiate oxidative stress and be cleared away from an organism by SOD. Then, TGP7 can serve as $O_{2}^{-}$· scavengers to get rid of radical injury together with SOD in cells.

#### 2.4.2. Relationship among Molecular Size, Amino Acid Composition, and Antioxidant Activity

Structural properties offer guidance for speculating the bioactivities of peptides and predicting their potential applications. To date, most reports suggested that MW, hydrophobicity, and the composition and sequence of amino acids were affecting the antioxidant ability of APs [14,50]. The three APs (TGP5, TGP7, and TGP9) from the gelatin hydrolysate of skipjack tuna scales are hexapeptide to octapeptide with MWs ranging from 608.57 Da to 804.92 Da (Table 5), which confirms that TGP5, TGP7, and TGP9 were easily contacted with target radical to play their antioxidant functions. 

Due to the hydrophobic properties of polyunsaturated fatty acids (PUFAs), amino acids with a hydrophobic branched chain have a high reactivity to them for helping them avoid the radical damage [47,51,52]. Furthermore, aromatic groups can stabilize radicals through donating protons to terminate the oxidative stress reaction [44,53]. Consequently, hydrophobic/aromatic amino acids, such as His, Pro, Met, Leu, and Phe, are regarded as the most important antioxidant factor of peptides [5,14]. For example, Jin et al. reported that the high antioxidant activities of MCLDSCLL and HPLDSLCL were duo to the amino acid residues of Leu, Met, and His in their sequences [54]. Therefore, His, Pro, and Hyp in the sequence of TGP5, Pro and His in the sequence of TGP7, and Met, Leu, Pro, and Phe in the sequence of TGP9 could significantly increase their antioxidant capacity. 

The hydrophobicity of peptides is important for accessibility to hydrophobic targets and enhances the affinity and reactivity of peptide. However, the composition and ratio of hydrophilic amino acids, such as Asp, Glu, Lys, and Gly, are pivotal in peptide antioxidant activity and are especially considered to be associated with antioxidant effects in vivo [55,56]. For example, the side chains of amino and carboxyl groups in amino acids are important for metal ion chelating and HO· scavenging activities of peptides [57,58,59]. Zhang et al. reported that Asp residues in WMFDW and Glu residues in EMGPA played critical roles in the activities of radical scavenging and lipid peroxidation inhibition [4]. Hu et al. reported that basic (Lys) and acidic (2Asp and Glu) amino acid residues in the sequence of NWDMEKIWD took responsibility for its outstanding activity [60]. In addition, Zhang et al. and Yang et al. reported that Gly residues could expediently donate hydrogen atoms to passivate active radicals because they could make the peptide backbone of gelatin and collagen more flexible [4,61]. Therefore, Gly and Glu in the amino acid sequence of TGP5, Asp, Lys, and 2Gly in the amino acid sequence of TGP7, and 2Gly in the amino acid sequence of TGP9 could play critical roles in their radical scavenging activities.

## 3. Experimental Section

### 3.1. Materials

Scales of skipjack tuna (*K. pelamis*) were supplied by Ningbo Todayfood Co. Ltd. (Ningbo, China). DEAE-52 cellulose and Sephadex G-25 were purchased from Shanghai Source Poly Biological Technology Co., Ltd (Shanghai, China). Acetonitrile (ACN) and trifluoroacetic acid (TFA) were purchased from Thermo Fisher Scientific Co., Ltd (Shanghai, China). DPPH was purchased from Sigma-Aldrich (Shanghai, China) Trading Co., Ltd. (Shanghai, China). Type I collagen (TC) from the scales of skipjack tuna was prepared in our lab.

### 3.2. Preparation of Scale Gelatin (TG) and Gelatin Hydrolysate of Kipjack Tuna

The pretreatment of scales, including removing non-collagenous proteins and minerals, was performed according to a previous method [61]. Then, the gelatin was extracted from pretreated scales according to the method described by Yang et al. with a slight modification [4]. In brief, the extraction process of gelatin was performed in DW at 60 °C for 8 h with a solid-to-liquid ratio of 1:10 (*w*/*v*). Finally, the extracting solution was centrifuged at 12,000 g for 15 min to remove the solid residues. The supernatant, named TG, was collected and lyophilized.

The hydrolytic process of TG was performed following the previous methods [56,61]. The TG dispersions (1%, *w*/*v*) were hydrolyzed separately using five proteases in the hydrolysis parameters in Table 6. After 4-hour hydrolysis, the hydrolysate was heated at 90 °C for 20 min and centrifuged at 8000× *g* for 25 min at room temperature. The resulting supernatants were collected, freeze-dried, and kept at −20 °C. The alcalase-hydrolysate was referred to as TGH. 

### 3.3. Characterization of Gelatin (TG)

#### 3.3.1. Proximate Analysis

Moisture, ash, fat, and protein contents of the scales and gelatin were measured using the methods of AOAC with the method numbers of 950.46B, 920.153, 960.39 (a), and 928.08, respectively [62]. An amino acid analysis was performed according to the methods described by Zhao et al. [36].

#### 3.3.2. SDS-PAGE

Electrophoretic patterns of TG and TC were performed using 4% stacking gel and 7.5% separating gel [26]. The samples (10 μg proteins) were mixed with the sample loading buffer at a ratio of 4:1 (*v*/*v*) in the presence of β-ME, then applied to sample wells and electrophoresed at a constant voltage of 100 V. After about 4 h, the gel was fixed with 10% acetic acid and 50% (*v*/*v*) methanol for 0.5 h. The gel was stained for 3 h with a Coomassie blue R-250 solution and de-stained with 10% (*v*/*v*) acetic acid and 30% (*v*/*v*) methanol solution. A high MW marker was used to estimate the MWs of proteins. TC was used as a collagen standard.

#### 3.3.3. FTIR

The infrared spectra (450–4000 cm^−1^) of TG and TC were recorded in KBr disks with a Fourier transform IR spectrophotometer (Nicolet 6700, Thermo Fisher Scientific Inc., Waltham, MA, USA). The mixture with the sample-to-KBr ratio of 1:100 (*w*/*w*) was pressed into a disk for spectrum recording.

### 3.4. Isolation of Peptides from TGH

TGH was fractionated with 3 and 5 kDa MWCO membranes and three fractions, including TGH-I (MW <3 kDa), TGH-II (MW 3–5 kDa), and TGH-III (MW >5 kDa) were collected and lyophilized. An amount of 5.0 mL of TGH-I solution (40.0 mg/mL) was injected into a pre-equilibrated column (1.6 cm × 80 cm) of DEAE-52 cellulose with DW, and stepwise eluted with 150 mL DW and NaCl solution (0.1 M, 0.5 M, and 1.0 M, respectively) at a flow rate of 1.0 mL/min, respectively. Each eluate (5.0 mL) was monitored at 214 nm. Finally, four fractions (AC-I to AC-IV) were collected and lyophilized on their chromatographic peaks. An amount of 5.0 mL of AC-III solution (20.0 mg/mL) was separated on a column (2.6 cm × 160 cm) of Sephadex G-25 and eluted with DW at a flow rate of 0.6 mL/min. Each eluate (3.0 mL) was collected and monitored at 214 nm, and three subfractions (GC-I, GC-II, and GC-III) were collected and lyophilized. GC-III was purified using RP-HPLC with a Zorbax, SB C-18 column (4.6 × 250 mm, 5 µm) on an Agilent 1260 (Santa Rosa, CA, USA). The sample was eluated with a linear gradient of ACN (0%–50% in 0–35 min) in 0.1% TFA at a flow rate of 0.8 mL/min. Ten APs (TGP1 to TGP10) were isolated at an absorbance of 214 nm and lyophilized.

### 3.5. Amino Acid Sequence and MW Analysis

The amino acid sequences of three APs (TGP5, TGP7, and TGP9) were measured on an Applied Biosystems 494 protein sequencer (Perkin Elmer/Applied Biosystems Inc, Foster City, CA, USA). 

The MWs of three APs (TGP5, TGP7, and TGP9) were determined using a Q-TOF mass spectrometer (Micromass, Waters, Milford, MA, USA) coupled with an electrospray ionization (ESI) source. Ionization was carried out in positive mode with a capillary voltage of 3500 V. Nitrogen was maintained at 40 psi for nebulization and 9 L/min at 350 °C for evaporation temperature. Data were collected in centroid mode from 100 to 2000 *m/z*.

### 3.6. Antioxidant Activity

The DPPH·, HO·, and $O_{2}^{-}$· scavenging assays of three APs (TGP5, TGP7, and TGP9) were determined by the previous method [56], and the half elimination ratio (EC_50_) was defined as the concentration where a sample caused a 50% decrease of the initial radical concentration.

#### 3.6.1. DPPH· Scavenging Activity

An amount of 2.0 mL of samples consisting of distilled water and different concentrations of the analytes were placed in cuvettes, and 500 μL of an ethanolic solution of DPPH (0.02%) and 1.0 mL of ethanol were added. A control sample containing the DPPH solution without the sample was also prepared. In the blank, the DPPH solution was substituted with ethanol. The DPPH· scavenging activity was calculated using the following formula:

DPPH· scavenging activity (%) = (*A*_c_ + *A*_b_ − *A*_s_)/*A*_c_ × 100%,

where *A*_s_ is the absorbance rate of the sample, *A*_c_ is the control group absorbance, and *A*_b_ is the blank absorbance.

#### 3.6.2. HO·Scavenging Activity

An amount of 1.0 mL of a 1.865 mM 1,10-phenanthroline solution and 2.0 mL of the sample were added to a screw-capped tube and mixed. Then, 1.0 ml of a FeSO_4_·7H_2_O solution (1.865 mM) was added to the mixture. The reaction was initiated by adding 1.0 mL of H_2_O_2_ (0.03%, *v/v*). After incubating at 37 °C for 60 min in a water bath, the absorbance of the reaction mixture was measured at 536 nm against a reagent blank. The reaction mixture without any antioxidant was used as the negative control and a mixture without H_2_O_2_ was used as the blank. The HO· scavenging activity was calculated using the following formula:

HO· scavenging activity (%) = [(*A*_s_ − *A*_n_)/(*A*_b_ − *A*_n_)] × 100%,

where *A*_s_, *A*_n_, and *A*_b_ are the absorbance values determined at 536 nm of the sample, the negative control, and the blank after the reaction, respectively.

#### 3.6.3. $O_{2}^{-}$· Scavenging Activity

Superoxide anions were generated in 1.0 mL of nitrotetrazolium blue chloride (NBT) (2.52 mM), 1.0 mL of NADH (624 mM) and 1 mL of different sample concentrations. The reaction was initiated by adding 1.0 mL of phenazine methosulphate (PMS) solution (120 μM) to the reaction mixture. The absorbance was measured at 560 nm against the corresponding blank after a 5-min incubation at 25 °C. The $O_{2}^{-}$· scavenging activity was calculated using the following equation:

$$O_{2}^{-}\cdot \;\mathrm{scavenging}\;\mathrm{activity}\;(\%)\;=\;[(A_{c}\;-\;A_{s})/A_{c}]\;\times \;100\%,$$

where *A*_c_ is the absorbance without sample and *A*_s_ is the absorbance with sample.

### 3.7. Statistical Analysis

The data are expressed as the mean ± SD (*n* = 3). An ANOVA test using the SPSS 19.0 (Statistical Program for Social Sciences, SPSS Corporation, Chicago, IL, USA) was used to comparatively analyze the mean value of each treatment. Duncan′s multiple range test was carried out to analyze the significant differences of samples (*p* < 0.05). 

## 4. Conclusions

In this study, the scale gelatin (TG) of skipjack tuna was prepared using a hot water extraction method and its physicochemical properties, including SDS-PAGE, FTIR, and amino acid composition, were systematically analyzed. The data indicate that the structure of TG was more unstable than that of TC and was suitable for preparation of gelatin hydrolysate. Therefore, TG was separately hydrolyzed under five proteases and three APs (TGP3, TGP5, and TGP9) with high antioxidant activity were isolated from the alcalase-hydrolysate and identified as His-Gly-Pro-Hyp-Gly-Glu (TGP5), Asp-Gly-Pro-Lys-Gly-His (TGP7), and Met-Leu-Gly-Pro-Phe-Gly-Pro-Ser (TGP9), respectively. The three APs (TGP3, TGP5, and TGP9) exhibited a high radical scavenging activity and may serve as antioxidant ingredients applied in new functional foods in the future.

## Figures and Tables

**Table 1:** Chemical composition of scales and scale gelatin (TG) of skipjack tuna.

Sample	Proximate Compositions (g/100 g of Dry Scale Weight)				Yield (%, Dry Weight Basis)
	Moisture	Fat	Ash	Protein	
Scales	28.37 ± 0.18	6.26 ± 0.29	47.61 ± 3.14	19.43 ± 1.08	
Gelatin (TG)	3.78 ± 0.39	0.53 ± 0.22	1.05 ± 0.16	94.08 ± 4.52	3.46 ± 0.27

All values are mean ± standard deviation (SD) (*n* = 3).

**Table 2:** Amino acid compositions of scale gelatin (TG) and type I collagen (TC) of skipjack tuna (residues/1000 residues).

Amino Acid	TG	TC
Hydroxyproline (Hyp)	80.7 ± 4.2	85.1 ± 2.4
Glutamic acid (Glu)	71.5 ± 3.5	75.9 ± 3.3
Aspartic acid (Asp)	44.2 ± 3.2	45.7 ± 2.1
Threonine (Thr)	24.3 ± 1.8	28.4 ± 0.8
Serine (Ser)	37.1 ± 1.8	39.2 ± 0.9
Proline (Pro)	113.4 ± 4.5	115.5 ± 3.4
Glycine (Gly)	327.9 ± 5.2	330.6 ± 4.6
Alanine (Ala)	125.1 ± 2.9	119.7 ± 2.7
Cysteine (Cys)	ND	0.0
Valine (Val)	19.8 ± 1.7	21.5 ± 0.7
Methionine (Met)	10.5 ± 0.8	6.1 ± 0.3
Isoleucine (Ile)	13.1 ± 0.8	11.4 ± 0.5
Leucine (Leu)	27.0 ± 1.2	23.4 ± 0.4
Tyrosine (Tyr)	7.2 ± 0.3	3.7 ± 0.5
Phenylalanine (Phe)	9.4 ± 0.8	3.3 ± 0.6
Hydroxylysine (Hyl)	6.4 ± 0.4	7.7 ± 0.4
Lysine (Lys)	24.8 ± 1.1	26.5 ± 1.1
Histidine (His)	8.5 ± 0.3	5.3 ± 0.3
Arginine (Arg)	47.1 ± 2.1	51.0 ± 1.4
Total	1000.0	1000.0
Imino acid (Pro + Hyp)	196.1	200.6

All values are mean ± standard deviation (SD) (*n* = 3). ND = not detected.

**Table 3:** The wavenumber of five major peaks (Amide A, B and I-III) of scale gelatin (TG) and type I collagen (TC) of skipjack tuna.

	Amide A	Amide B	Amide I	Amide II	Amide III
TC (cm^−1^)	3325	2977	1689	1484	1209
TG (cm^−1^)	3351	2939	1667	1536	1215

**Table 4:** Degree of hydrolysis (DH, %) and hydroxide radical (HO·) scavenging activity (%) of the gelatin hydrolysate of skipjack tuna scales using five kinds of proteases.

Protease	DH (%)	HO·Scavenging Activity (5.0 mg/mL, %)
Pepsin	18.39 ± 1.12 ^a^	19.21 ± 1.25 ^a^
Papain	15.39 ± 0.84 ^a^	16.58 ± 0.93 ^a^
Trypsin	19.64 ± 0.96 ^b^	20.87 ± 2.33 ^b^
Neutrase	21.67 ± 1.34 ^b^	23.72 ± 1.08 ^b^
Alcalase	25.35 ± 1.68 ^c^	29.46 ± 1.37 ^c^

The data are presented as the mean ± SD (*n* = 3). ^a–c^ Values with same letters in each column indicate no significant difference (*p* > 0.05).

**Table 5:** Amino acid sequences, average MWs and radical scavenging activities of three isolated peptides (TGP5, TGP7, and TGP9) from gelatin hydrolysate of skipjack tuna (*K. pelamis*) scales.

	Amino Acid Sequence	Theoretical Mass/Observed Average Mass (Da)	EC_50_ (mg/mL) ^a^		
			DPPH·	HO·	$O_{2}^{-}$·
TGP5	His-Gly-Pro-Hyp-Gly-Glu	608.60/608.57	1.34	1.03	1.19
TGP7	Asp-Gly-Pro-Lys-Gly-His	609.63/609.61	0.54	0.41	0.71
TGP9	Met-Leu-Gly-Pro-Phe-Gly-Pro-Ser	804.95/804.92	0.67	0.74	1.59

**Table 6:** Hydrolysis parameters of different proteases.

Protease	Temperature (°C)	Enzyme Dosage (g /100 g Scale)	Time (h)	pH Value
Pepsin	37	2	4	2.0
Papain	50	2	4	6.0
Trypsin	37	2	4	7.0
Neutrase	60	2	4	7.0
Alcalase	50	2	4	8.0
